# Spotlight on researchers during a wet-lab to dry-lab transition period: an interview with Guifen Liu and Qi Wang

**DOI:** 10.1038/s42003-024-06923-x

**Published:** 2024-10-08

## Abstract

Dr. Guifen Liu, now an Associate Professor at ZhangLab, Tongji University, began her research in epigenetic regulation using zebrafish as a model, first as a postdoctoral researcher and then as a research scientist. She established the zebrafish culturing system at the Department of Informatics, Tongji University, Shanghai, China. Dr. Qi Wang is an Assistant Professor at ZhangLab, Tongji University, and she is in charge of the cell culture part of the lab and has broad expertise in high-throughput experimental research on chromatin structure. They are the only two experimental biologists in a dry lab focusing on different research topics. At present, they are in the transition period from experimental scientists to computational scientists. Current Lab Members-Yong Zhang Lab (tongji.edu.cn).

**Figure:**
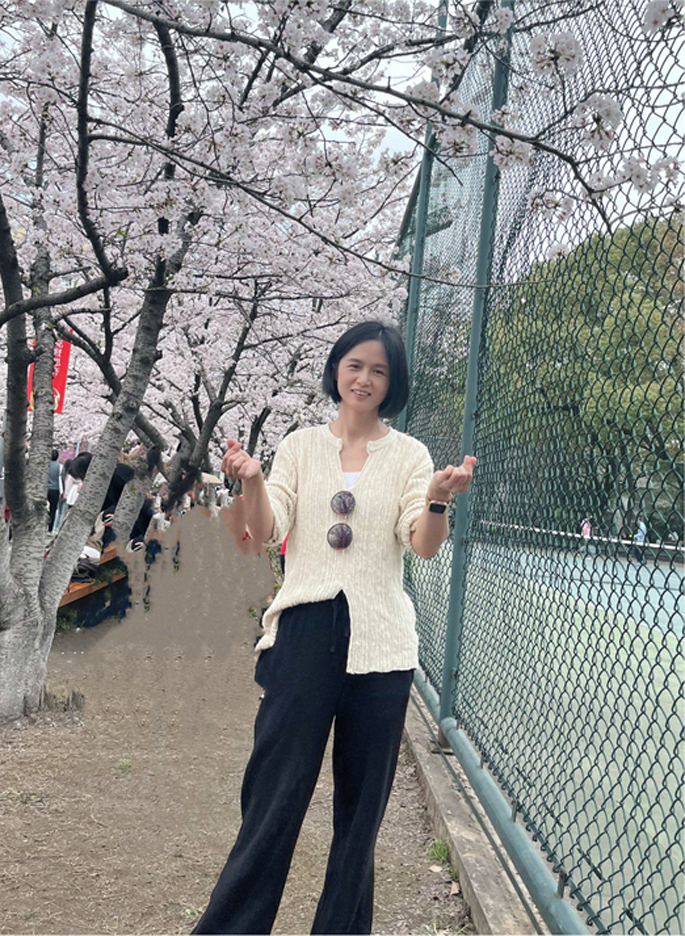
Jingfan Zhu. Pictured: Guifen Liu

Please tell us about the journey of your career.

Guifen Liu **[GL]** In my professional journey, I have been fortunate to experience a series of research stages that are both independent and interconnected. During my master’s program, my research focused on identifying molecular targets in gastrointestinal tumors, which is a basic and critical starting point. As my understanding of epigenetics deepened, I was drawn to the immense potential of this field. Consequently, I chose to continue my studies for a PhD and to delve into the study of epigenetic regulation mechanisms in eukaryotes. There, my previous group was dedicated to traditional wet lab experiments, which provided me with valuable experimental skills and rigorous scientific training.

However, with the explosive growth of -omics data, I realized the enormous potential of combining wet lab experiments with computational biology. By chance, I learned that my current research group was recruiting postdoctoral fellows. Without hesitation, I joined them and embarked on a journey of integrated dry and wet lab research. In summary, my scientific career has evolved with my deepening understanding of the field and societal development. This process has not only broadened my research horizons but also reinforced my belief in the importance of lifelong learning.

Qi Wang **[QW]** My career path is quite traditional: I earned a bachelor’s degree in biology, followed by a master’s, a PhD, and then postdoctoral training, eventually finding a position at a university. This route feels natural and requires little thought, as many people have followed it. Besides, the life sciences are inherently fascinating. I’ve been involved in research on chromatin structure, which is a very traditional field, yet there are always new discoveries that amaze you and highlight the intricacies of life.

Can you, as an experimental scientist, tell us the differences of working in a “dry” lab from a “wet” lab?

**[GL]** First and foremost, I believe that whether it is in a dry lab or a wet lab, the core objective of research remains the same: to deeply understand and reveal the laws governing the development of life phenomena. Although there are differences in research methods and the technical means employed, they each play an indispensable role. In the wet lab, direct experimental operations are necessary to observe and verify biological phenomena; this process often requires more time and resources and involves repeated trials, attempts, and optimizations until reliable results are obtained. Therefore, the experimental results obtained by this method are considered to be more reliable and precise. In contrast, research in the dry lab focuses on computational simulations and the analysis of large-scale datasets, which can easily test and verify a variety of hypotheses, saving time and labor to some extent. The efficiency of the dry lab lies in its ability to handle complex data and build predictive models, providing new perspectives and solutions for biological research. The predictions obtained through computational methods in the dry lab are further verified by experiments in the wet lab. This integrated approach of combining dry and wet research greatly improves the efficiency of research. Therefore, the combination of dry and wet lab research is complementary. This interdisciplinary research method provides us with a comprehensive and three-dimensional perspective, enabling us to more fully understand the complexity of life sciences.

**[QW]** Strictly speaking, I haven’t worked in a completely traditional wet lab. During my PhD, I was in a “mixed” lab where my advisor came from a physics background, and the lab included pure experimentalists, data analysts, and researchers in biophysics and engineering. I appreciate inclusive environments with diverse perspectives, which is one of the reasons I ultimately chose to join a dry lab. In a dry lab conducting wet experiments, there might not be many colleagues to discuss details with when specific issues arise. However, the advantage is gaining a completely different perspective on problem-solving and data interpretation. Personally, I see wet experiments as more of a bottom-up approach, where we tend to design experiments based on the questions we’re investigating and let the results drive our next steps. In contrast, dry experiments resemble a top-down approach, where we might skip intermediate steps to consider what types of data are needed to address a specific problem, allowing for a more comprehensive planning of the work.

With more and more research being interdisciplinary, can you speak about the potential advantage of having seen both sides of a lab, wet and day?

**[GL]** The complexity of biological issues often requires analysis from multiple levels and perspectives. The most significant advantage of researchers with experience in both dry and wet labs is the ability to combine the intuitiveness of experimental observations with the precision of data analysis, thereby providing a comprehensive perspective to understand these complex problems. This integrated capability enables us to design or develop new research methods, propose effective solutions, and quickly determine which method is more effective in a given context, thereby significantly improving research efficiency.

Another advantage of interdisciplinary research experience is the ability to adapt to new research directions. In today’s rapid development of artificial intelligence and deep learning, having an interdisciplinary background allows us to quickly master and apply these technologies in biological research. For example, we can build pre-trained large models to predict biological events, bringing breakthrough progress to the research field. Interdisciplinary experience keeps us highly sensitive to emerging technologies, enabling us to foresee technological trends and plan in advance how to integrate these technologies into our research. The combination of artificial intelligence and robotics, for example, can revolutionize research thinking and methods. Moreover, interdisciplinary experience makes it easier for us to establish cooperative relationships with researchers in different fields and explore new research areas.

**[QW]** I believe that as interdisciplinary research grows and both dry and wet experiments evolve, the distinction between them may blur. With technological advancements, wet experiments are likely to adopt more data-driven approaches and deep learning models in their design. Additionally, many wet lab techniques can increasingly rely on engineered robots or other automated methods rather than solely manual processes. In this scenario, dry labs will find it easier to validate their conclusions derived from data through experimental approaches during project design and scientific exploration.

Can you speak of any challenges that you are facing?

**[GL]** My research focuses on the integration of computational biology and high-throughput omics data, with the goal of understanding the complex mechanisms of genomic transcriptional activation. With the rise of large models, our field has encountered new opportunities and challenges. Currently, the main challenge I face is how to effectively integrate large models into the existing research framework, which requires interdisciplinary knowledge and skills. I first need to learn the basic knowledge and methods of machine learning and deep learning. This includes but is not limited to in-depth learning of model structures such as Multilayer Perceptron (MLP), Convolutional Neural Networks (CNN), Graph Neural Networks (GNN), as well as Transformer architectures and attention mechanisms. Through the accumulation of this knowledge, I will be able to have a deeper understanding of the prediction results of large models and how to connect these results to deep biological mechanisms.

In addition, I am also facing the challenge of applying for project funding. Considering the latest changes in the national natural science fund policy of our country, the increase in the number of applicants has indeed increased the difficulty of application. Therefore, I need to actively accumulate research foundations and preliminary results, including publishing research results in peer-reviewed academic journals and participating in key academic conferences to enhance my academic influence and visibility. Of course, the application for scientific research funding is a process full of uncertainty. Even in the face of rejection, I will maintain a positive attitude, carefully analyze the review comments, learn valuable opinions from them, and improve the possibility of obtaining funding.

**[QW]** There are indeed many challenges. The pace of progress in the field seems to be accelerating, which creates a sense of urgency and anxiety that operates in the background, making it difficult to relax fully. And a significant part of the challenge stems from external evaluation systems that set the standards for what grants and publications you should have. This system functions as a feedback loop: obtaining funding leads to a positive cycle where you can hire students, carry out projects as scheduled, and achieve good results, enabling you to apply for more fundings. Conversely, without funding, it feels like an uphill struggle. The immediate result of insufficient funds is the inability to have students, leading to understaffing and slow progress, which in turn creates a series of negative feedback effects.

What advice would you give to a young female biologist?

**[GL]** First and foremost, never stop the pursuit of learning. In the rapidly evolving field of biology, continuous learning is crucial. With the continuous emergence of new technologies and theories, maintaining curiosity and the desire to learn will help us keep up with the times. Whether through reading literature or attending academic conferences, you must constantly enrich yourself and update your knowledge base. Learning is not limited to our professional field. Cross-field and interdisciplinary knowledge can often inspire innovative thinking. Only through continuous learning can we avoid being replaced by artificial intelligence and robots in the near future.

Secondly, never forget why you started. On the journey of scientific research, you may encounter many challenges and pressures, but please do not forget the initial passion that inspired you to choose biology. When facing difficulties, recall these motivations, they will give you strength and inspire you to keep moving forward. Do not fear failure, be brave in trying, even if the results are not as expected, the experience gained in the process is invaluable. Step out of your comfort zone and dare to challenge yourself, and you will find your potential is far beyond what you imagined.

Lastly, maintain physical and mental health. Scientific research often requires a high degree of focus and dedication, but do not forget to take care of your well-being. There is an old Chinese saying, “Actions speak louder than words,” and I believe your attitude towards learning and your unwavering efforts not only bring positive energy to your family and children but also inspire them to learn and explore bravely.

**[QW]** In my view, establishing a reliable self-support system is essential, both in academic work and in life and personal health. Academic research inevitably comes with challenges and bottlenecks, and it’s wonderful if you can find joy in tackling these obstacles. However, during times of significant pressure, engaging in activities that bring you happiness—such as exercise, hobbies, or family time—can help balance the stress of work and provide a sense of accomplishment in other areas. I think this is a sustainable approach to work and life.

**Figure:**
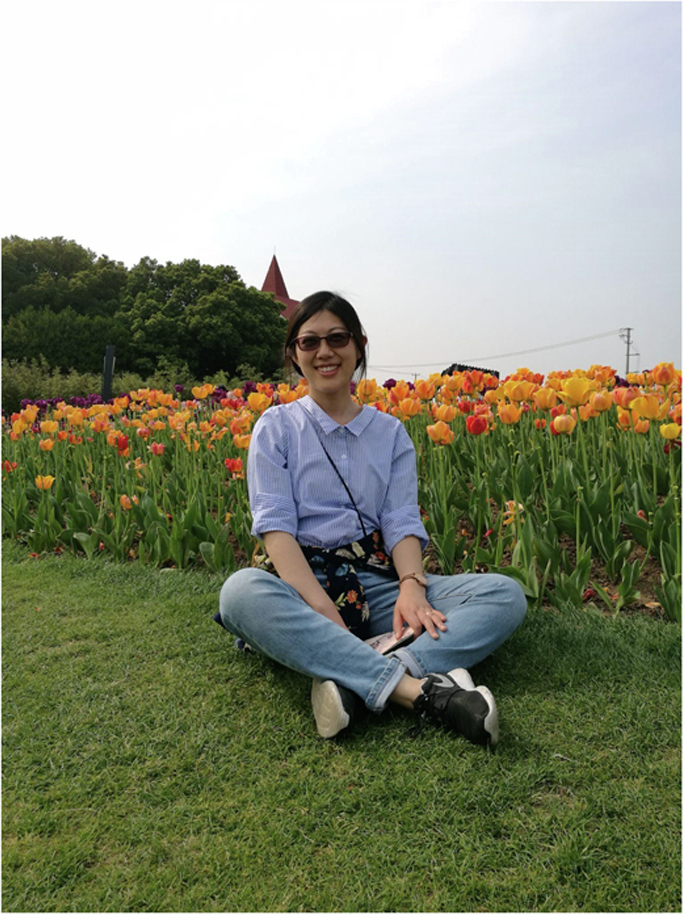
Yang Li. Pictured: Qi Wang

*This interview was conducted by Associate Editor Mengtan Xing*.

